# Author Correction: Demonstration of quantum-digital payments

**DOI:** 10.1038/s41467-023-40866-x

**Published:** 2023-08-21

**Authors:** Peter Schiansky, Julia Kalb, Esther Sztatecsny, Marie-Christine Roehsner, Tobias Guggemos, Alessandro Trenti, Mathieu Bozzio, Philip Walther

**Affiliations:** 1 University of Vienna, Faculty of Physics, Vienna Center for Quantum Science and Technology (VCQ), 1090 Vienna, Austria; 2 Christian Doppler Laboratory for Photonic Quantum Computer, Faculty of Physics, University of Vienna, 1090 Vienna, Austria; 3 Present Address: Security and Communication Technologies, Center for Digital Safety and Security, AIT Austrian Institute of Technology GmbH, Giefinggasse 4, 1210 Vienna, Austria

**Keywords:** Quantum information, Quantum optics

Correction to: *Nature Communications* 10.1038/s41467-023-39519-w, published online 29 June 2023

The original version of this Article contained an error in the Discussion section, 4^th^ paragraph, 6^th^ sentence, which incorrectly read ‘Remarkably, we can amend our protocol such that the number of purchases is not bounded by the MAC function: the Client simply encrypts a new random key *R* with *C*, and forwards the result along with the cryptogram to the TTP.’ The correct version is ‘We can amend our protocol such that the number of purchases is not bounded by the MAC function, by growing *C* during the payment process: when the Client receives a new quantum token |*P*〉, we append additional quantum states for QKD, and use the cryptogram *κ* for authentication.’

This has been corrected in both the PDF and HTML versions of the Article.

