# Supplementary material for: Demonstration of quantum-digital payments
Source: Nat Commun. 2023 Jun 29;14:3849. doi: 10.1038/s41467-023-39519-w (PMC10310712; doi:10.1038/s41467-023-39519-w)
Supplement: Supplementary file 1 — Supplementary Information [file 41467_2023_39519_MOESM1_ESM.pdf]

# Supplementary Information for "Demonstration of quantum-digital payments"

Peter Schiansky,[1,*] Julia Kalb,[1,*] Esther Sztatecsny,[1] Marie-Christine Roehsner,[1,†] Tobias Guggemos,[1] Alessandro Trenti,[1,†] Mathieu Bozzio,[1,‡] and Philip Walther[1,2,‡]

[1] *University of Vienna, Faculty of Physics, Vienna Center for Quantum Science and Technology (VCQ), 1090 Vienna, Austria*
[2] *Christian Doppler Laboratory for Photonic Quantum Computer, Faculty of Physics, University of Vienna, 1090 Vienna, Austria*

Supplementary Note 1 introduces the mathematical tools required to understand our security analysis, namely semidefinite programming, Choi's theorem on completely positive maps and Chernoff-Hoeffding bounds. Supplementary Note 2 introduces the cryptographic tools required to understand the classical security analysis, namely hash functions and message authentication codes (MAC and HMAC), as well as their security properties. Supplementary Note 3 derives the information-theoretic security against double-spending, while Supplementary Note 4 proves the information-theoretic security of our cryptogram, concealing the secure key $C$. Following the formal security analyses, Supplementary Note 5 provides some intuition as to why no authenticated channel is required after the initial secure sharing of $C$. Supplementary Note 6 finally provides our protocol performance along with detailed setup characterizations.

## Supplementary Note 1. MATHEMATICAL PRELIMINARIES

### A. Semidefinite programming

Quantum theory relies on linear algebra. In quantum cryptography, security analyses often involve optimizing over semidefinite positive objects to find the adversary's optimal cheating strategy. Most of the time, these objects are density matrices, measurement operators, or more general completely positive trace-preserving (CPTP) maps. Semidefinite programming provides a suitable framework for this, as it allows to optimize over semidefinite positive variables, given linear constraints [1–3].

A semidefinite program may be defined as a triple $(\Lambda, F, C)$ where $\Lambda$ is a Hermitian-preserving CPTP map, and $F$ and $C$ are Hermitian operators living in complex Hilbert spaces $\mathcal{H}_F$ and $\mathcal{H}_C$, respectively. We start by defining a maximization problem, which will serve as our *primal problem.* The primal problem maximizes a *primal objective function*, $\mathrm{Tr}\left(F^\dagger X\right)$, over all positive semidefinite variables $X$, given a set of linear constraints expressed as a function of $C$:

$$\begin{aligned} \text{maximize} \quad & \mathrm{Tr}\left(F^\dagger X\right) \\ \text{s.t.} \quad & \Lambda(X) = C \\ & X \geqslant 0. \end{aligned} \tag{1}$$

If it exists, the operator $X$ which maximizes $\mathrm{Tr}\left(F^\dagger X\right)$ given these constraints is the *primal optimal solution*, and the corresponding value of $\mathrm{Tr}\left(F^\dagger X\right)$ is the *primal optimal value.*

Semidefinite programs present an elegant dual structure, which associates a dual minimization problem to each primal maximization problem. Effectively, the new variable(s) $Y$ of the dual problem may be understood as the Lagrange multipliers associated with the constraints of the primal problem (one for each constraint). The dual problem associated with (1) reads [1–3]:

---

* These two authors contributed equally.
† Current address: Security and Communication Technologies, Center for Digital Safety and Security, AIT Austrian Institute of Technology GmbH, Giefinggasse 4, 1210 Vienna, Austria.
‡ Corresponding authors: mathieu.bozzio@univie.ac.at, philip.walther@univie.ac.at.

$$\begin{aligned} \text{minimize} \quad & \operatorname{Tr}\left(C^\dagger Y\right) \\ \text{s.t.} \quad & \Lambda^*(Y) - F \geqslant 0 \\ & Y = Y^\dagger. \end{aligned} \tag{2}$$

Similarly to the primal problem, the operator $Y$ which minimizes $\operatorname{Tr}\left(C^\dagger Y\right)$ given these constraints, if it exists, is the *dual optimal solution*, and the corresponding value of $\operatorname{Tr}\left(C^\dagger Y\right)$ is the *dual optimal value.*

The Lagrange multiplier method allows to find the local extremum of a constrained function. The optimal value $s_p$ of the primal problem therefore lower bounds the optimal value $s_d$ of the dual problem, while the optimal value of the dual upper bounds that of the primal. This property is known as *weak duality*, and may be simply expressed as:

$$s_p \leqslant s_d. \tag{3}$$

In many quantum-cryptographic applications however, we wish to ensure that the upper bound derived in the primal problem is *tight*, i.e. that the local maximum is in fact a global maximum for the objective function. The dual problem will help to prove this when there exists *strong duality*:

$$s_p = s_d. \tag{4}$$

### B. Choi's theorem on completely positive maps

Let us consider a tensor product of two $d$-dimensional Hilbert spaces $\mathcal{H} = \mathcal{H}_1^d \otimes \mathcal{H}_2^d$, and then define the maximally entangled state $|\Phi^+\rangle\langle\Phi^+|$ on $\mathcal{H}$ as:

$$|\Phi^+\rangle\langle\Phi^+| = \frac{1}{d}\sum_{i,j=1}^{d} |i\rangle\langle j| \otimes |i\rangle\langle j|. \tag{5}$$

We introduce a completely positive linear map $\Lambda : \mathcal{H}_1^d \to \mathcal{H}_3^{d'}$, and define the Choi-Jamiolkowski operator $J(\Lambda)$ : $\mathcal{H}_1^d \otimes \mathcal{H}_2^d \to \mathcal{H}_3^{d'} \otimes \mathcal{H}_2^d$ as the operator which applies $\Lambda$ to the first half of the maximally entangled state $|\Phi^+\rangle\langle\Phi^+|$:

$$J(\Lambda) = \frac{1}{d}\sum_{i,j=1}^{d} \Lambda(|i\rangle\langle j|) \otimes |i\rangle\langle j|. \tag{6}$$

Choi's theorem then states that $\Lambda$ is completely positive if and only if $J(\Lambda)$ is positive semidefinite. We also have that $\Lambda$ is a trace-preserving map if and only if $\operatorname{Tr}_{\mathcal{H}_3^{d'}}(J(\Lambda)) = \mathbb{1}_{\mathcal{H}_2^d}$ [1–3].

### C. Chernoff-Hoeffding bounds

Chernoff-Hoeffding inequalities provide exponentially decreasing bounds on tail distributions of sums of independent random variables [4–6].

Let us assume a set of $N$ random variables $X_i$, which can each take the value 1 with probability $p_i$ and 0 with probability $1 - p_i$. The probability that $X = \sum_{i=0}^{N-1} X_i$ is larger than the expected mean value $\mu$ by some $\delta \in [0, 1]$ is bounded by the following *upper tail*:

$$P[X \geq (1+\delta)\cdot\mu] \leq \exp\left(-\frac{\delta^2}{3}\cdot\mu\cdot N\right) \tag{7}$$

Similarly, the probability that $X = \sum_{i=0}^{N-1} X_i$ is smaller than the expected mean value by some $\delta \in [0, 1]$ is bounded by the *lower tail*:

$$P[X \leq (1-\delta)\cdot\mu] \leq \exp\left(-\frac{\delta^2}{2}\cdot\mu\cdot N\right) \tag{8}$$

## Supplementary Note 2. CRYPTOGRAPHIC PRELIMINARIES

We first describe the preliminaries of hash functions, *MAC*s and H*MAC*s in a computationally-secure setting (such that attacks are bounded by polynomial time attackers). We then show how to construct an *i.t.-secure* H*MAC* with these ingredients and define its security bounds against *unbounded* attackers. The interested reader is referred to [7, 8] for further reading.

### A. Hash function

A hash function is defined as a function that maps a set of arbitrary length to a finite set $H : \{0,1\}^* \mapsto \{0,1\}^n; n \in \mathbb{N}$. Hence, a hash function $H$ is non-injective by definition, and threatened by:

- **collision attacks:** identifying two different arbitrary inputs $x_1$ and $x_2$ such that $H(x_1) = H(x_2)$,
- **second pre-image attacks:** finding a pre-image input $x_2$, such that $H(x_1) = H(x_2)$, given an input string $x_1$,
- **pre-image attacks:** for a given output string $y$, finding a pre-image input $x$, such that $y = H(x)$.

If $H$ withstands collision attacks, we call it *collision-resistant*; in that case, $H$ is also *second pre-image* and *pre-image* resistant. We consider $H$ to be a *secure* or *cryptographic* hash function, if it is *collision-resistant* against computationally bounded attacks.

### B. Message Authentication Code (MAC)

A *MAC* is a function that takes a key $k \in \{0,1\}^n; n \in \mathbb{N}$ and message $m \in \{0,1\}^*$ as input and subsequently outputs a tag $y \in \{0,1\}^*$ such that $MAC(k,m) \mapsto y$. Upon receiving the tuple $(y,m)$, an honest verifier in possession of $k$ can verify the authenticity of a message, i.e., $y = MAC(k,m)$.

We consider a *MAC* to be secure if *existential unforgeability against a chosen message attack* holds, where:

- **existentially unforgeable** means that the attacker is not able to generate a valid MAC tag on any message, without being in possession of the key $k$,
- **chosen message attack** means that the attacker is able to obtain *MAC* tags on any other messages before performing the attack.

### C. HMAC

A typical implementation of a *MAC* is the so called *Keyed-Hash Message Authentication Code (HMAC)*, which is a function $f(H,k,m) \mapsto y$. Based on a hash function $H$, it takes a secret key $k$ and message $m$ as inputs, and outputs some authentication tag. The *HMAC* under the use of the hash function $H$ is defined as:

$$HMAC(k,m) = H\big(k \oplus \text{opad} \parallel H\,(k \oplus \text{ipad} \parallel m)\,\big),$$

where $\oplus$ is a bitwise XOR; $\parallel$ means simple concatenation; and `opad` and `ipad` are fixed public strings with a length depending on the underlying function of $H$.

We consider an *HMAC* to be a secure MAC function (i.e., *existentially unforgeable against a chosen message attack*), if the underlying hash function $H$ is *collision resistant.*

### D. i.t.-secure MAC

A *Message Authentication Code* (MAC) is called *information-theoretically* or *perfectly* or *unconditionally* secure if it is secure against [8–10]:

- **impersonation attacks**: where an attacker can create a message and a tag, valid under the key in use,

- **substitution attacks**: where an attacker sees one valid message-tag pair, intercepts it, and then replaces it with another valid message-tag pair.

In other words, there is no $MAC(k,m) = MAC(k,m')$ for $m \neq m'$ (given that $k$ remains secret).

To construct such a function, we may for example consider an *authentication matrix* $\mathcal{T}$, that is constructed with a *strongly universal* keyed function $h(\mathcal{K},\mathcal{M})$ for the keyspace $\mathcal{K}$ and an arbitrary input string $\mathcal{M}$, i.e. $\mathcal{T} = |\mathcal{K}| \times |\mathcal{M}|$. Typical examples for $h$ were studied in [9, 10], but it can also be a computationally secure MAC or HMAC function.

To generate an authentication tag for a message $m \in \mathcal{M}$ and a given key $k \in \mathcal{K}$, one takes the corresponding cell $t \in \mathcal{T}$ as an out put. Assuming a uniform distribution of $\mathcal{K}$ and generating a new $\mathcal{T}$ for every message $m \in \mathcal{M}$, the probability of forging a valid authentication tag is $p_t = 1/|\mathcal{T}|$ if the key is only used once.

The tag space $|\mathcal{T}|$ depends on the message- and key space. For a message space of size $|\mathcal{K}| = |\mathcal{M}|^2$, the probability of forging a valid authentication tag is

$$p_t = \frac{1}{|\mathcal{T}|} = \frac{|\mathcal{M}|}{|\mathcal{K}|} = \frac{1}{\sqrt{|\mathcal{K}|}}$$

.

A construction as above is referred to *1-time-secure*, whereas similar other *n-time-secure* constructions exist if $k \in \mathcal{K}$ is to be used multiple times [11, 12].

Note that, while assuming such a matrix is not necessary for ITS authentication, we use it as an example to simplify the above cheating probability derivation.

## Supplementary Note 3. SECURITY AGAINST DOUBLE-SPENDING

### A. For $N = 1$

This section derives the security analysis for a token consisting of $N = 1$ quantum state. The aim is to derive a border between the secure region of operation, containing all pairs of experimental imperfections $(l, e)$ for which the presence of a malicious party can be detected, and its corresponding insecure region, containing all pairs of dishonest experimental deviations $(l, e)$ for which a malicious behavior cannot be detected. In both cases, $l$ denotes the fraction of quantum states from $|P\rangle$ that are declared as losses, while $e$ denotes the fraction of quantum states from $|P\rangle$ for which the declared measurement outcome disagrees with the classical description $(b, \mathcal{B})$.

In the simplest case, a successful attack consists in producing two cryptograms $\kappa_0$ and $\kappa_1$ for two distinct Merchants $M_0$ and $M_1$ that both pass the TTP's verification test. We note that in this two-merchant scenario, the information contained in the output of the HMAC function is one bit regardless of the actual length of the output. We may therefore reduce the commitments $M_0$ and $M_1$ to measurements of $|P\rangle$ in the $Z$ and $X$ bases, respectively.

In order to succeed in their optimal attack, the dishonest party may perform any general quantum operation on $|P\rangle$, and replace all lossy and noisy channels by perfect ones. The TTP may then detect an attack only if their (potentially tampered with) measured noise and losses lie within the secure region of operation. We use SDP techniques from Supplementary Note 1 A to minimize the errors $e$ that the adversary must induce/declare while introducing at most $l$ losses. We optimize over the set of all possible CPTP maps $\{\Lambda\}$, that produce two classical cryptograms living in Hilbert space $\mathcal{H}_0 \otimes \mathcal{H}_1$ from the original experimental quantum token state $\rho_P$ living in $\mathcal{H}_P$. The resulting secure/insecure regions of operation are shown in Supplementary Figure 1.a.

We note that $\mathcal{H}_0$ and $\mathcal{H}_1$ are 3-dimensional Hilbert spaces spanned by classical answers $\{|a_0\rangle, |a_1\rangle, |\varnothing\rangle\}$, where $|a_0\rangle$ and $|a_1\rangle$ are orthonormal basis vectors indicating two possible classical answers (0 and 1 respectively), and $|\varnothing\rangle$ is a third basis vector (orthogonal to the two others) indicating the declaration of a lost state. On the other hand, $\mathcal{H}_P$ is a 7-dimensional Hilbert space spanned by $\{|v\rangle, |q_0\rangle, |q_1\rangle, |m_0\rangle, |m_1\rangle, |m_2\rangle, |m_3\rangle\}$, where $|v\rangle$ is the vacuum state, $|q_0\rangle$ and $|q_1\rangle$ span a qubit space, and $|m_i\rangle$ constitute the four orthogonal outcomes which materialize the four perfectly distinguishable states in the multiphoton subspace. Since the states produced by SPDC are of the form $\sum_{n=0}^{\infty} c_n |n\rangle_1 |n\rangle_2$ in the $\{|n\rangle\}$ photon number basis [13], this leaves the individual subsystems in states of the form $\sum_{n=0}^{\infty} \widetilde{c}_n |n\rangle\langle n|$. Our four states may then be written as the following density matrices :

$$\begin{aligned}
\sigma_0 &= p_0|v\rangle\langle v| + p_1|+\rangle\langle +| \quad + p_m|m_0\rangle\langle m_0| \\
\sigma_1 &= p_0|v\rangle\langle v| + p_1|+i\rangle\langle +i| + p_m|m_1\rangle\langle m_1| \\
\sigma_2 &= p_0|v\rangle\langle v| + p_1|-\rangle\langle -| \quad + p_m|m_2\rangle\langle m_2| \\
\sigma_3 &= p_0|v\rangle\langle v| + p_1|-i\rangle\langle -i| + p_m|m_3\rangle\langle m_3|,
\end{aligned}$$

where $|+\rangle$,$|+i\rangle$, $|-\rangle$, $|-i\rangle$ are the usual $X$ and $Y$ eigenstates in the qubit space spanned by $|q_i\rangle$ and the photon number populations $p_n$ are estimated from our experiment. This allows to express the experimental quantum token state $\rho_P$ as:

$$\rho_P = \frac{1}{4}\sum_{k=0}^{3}\sigma_k. \tag{9}$$

The probability $P_0$ that $\kappa_0$ does not pass the TTP's verification is then given by:

$$P_0 = \mathrm{Tr}\sum_{k=0}^{3}\left(\frac{1}{2}\left|a_k^{\perp}\right\rangle\left\langle a_k^{\perp}\right| \otimes \mathbb{1}\right)\Lambda\left(\frac{1}{4}\sigma_k\right), \tag{10}$$

while the probability $P_1$ that $\kappa_1$ does not pass the TTP's verification reads:

$$P_1 = \mathrm{Tr}\sum_{k=0}^{3}\left(\mathbb{1}\otimes\frac{1}{2}\left|a_k^{\perp}\right\rangle\left\langle a_k^{\perp}\right|\right)\Lambda\left(\frac{1}{4}\sigma_k\right). \tag{11}$$

where $|a_k^{\perp}\rangle$ is the wrong, orthogonal answer to $|a_k\rangle$. Using Eq. (6), we may rewrite these expressions as $P_0 = \mathrm{Tr}\left(E_0 J(\Lambda)\right)$ and $P_1 = \mathrm{Tr}\left(E_1 J(\Lambda)\right)$, where $E_0$ and $E_1$ are the *error operators*:

$$\begin{aligned} E_0 =& \frac{1}{4}\sum_{k=0}^{3}\frac{1}{2}\left|a_k^{\perp}\right\rangle\left\langle a_k^{\perp}\right| \otimes \mathbb{1}\otimes\overline{\sigma_k}, \\ E_1 =& \frac{1}{4}\sum_{k=0}^{3}\mathbb{1}\otimes\frac{1}{2}\left|a_k^{\perp}\right\rangle\left\langle a_k^{\perp}\right|\otimes\overline{\sigma_k}. \end{aligned} \tag{12}$$

where $\overline{\sigma_k}$ denotes the complex conjugate of $\sigma_k$. Following a similar reasoning, the probability that the dishonest party declares losses for $\kappa_0$ (resp. 1) reads $\mathrm{Tr}\left(L_0 J(\Lambda)\right)$ (resp. $\mathrm{Tr}\left(L_1 J(\Lambda)\right)$), where $L_0$ and $L_1$ are the *loss operators*, containing the projection onto the state $|\varnothing\rangle$:

$$\begin{aligned} L_0 &= \frac{1}{4}\sum_{k=0}^{3}|\varnothing\rangle\langle\varnothing|\otimes\mathbb{1}\otimes\overline{\sigma_k}, \\ L_1 &= \frac{1}{4}\sum_{k=0}^{3}\mathbb{1}\otimes|\varnothing\rangle\langle\varnothing|\otimes\overline{\sigma_k}. \end{aligned} \tag{13}$$

We now search for the optimal CPTP map $\Lambda$ that minimizes $e$ for a fixed $l$. We recast this problem as the following primal SDP (which we choose to be a minimization problem rather than a maximization problem for the sake of intuition):

$$\begin{aligned} \min \quad & \mathrm{Tr}\left(E_0 J(\Lambda)\right) \\ \text{s.t.} \quad & \mathrm{Tr}_{\mathcal{H}_0\otimes\mathcal{H}_1}\left(J(\Lambda)\right) = \mathbb{1}_{\mathcal{H}_P} \\ & \mathrm{Tr}\left(E_0 J(\Lambda)\right) \geqslant \mathrm{Tr}\left(E_1 J(\Lambda)\right) \\ & \mathrm{Tr}\left(L_0 J(\Lambda)\right) \leqslant l \\ & \mathrm{Tr}\left(L_1 J(\Lambda)\right) \leqslant l \\ & J(\Lambda) \geqslant 0 \end{aligned} \tag{14}$$

The first constraint imposes that $\Lambda$ is trace-preserving, the second imposes that the error rate for cryptogram $\kappa_0$ is at least equal to that for cryptogram $\kappa_1$, the third and fourth impose that the losses declared for $\kappa_0$ and $\kappa_1$ do not exceed the expected honest losses, and the fifth imposes that $\Lambda$ is completely positive.

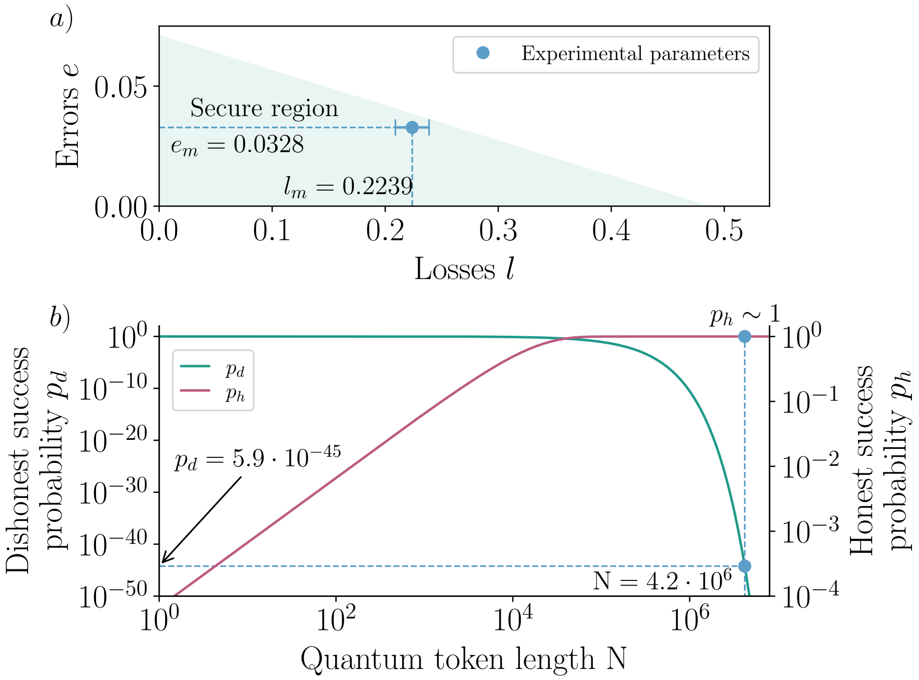


Supplementary Figure 1: **Security for experimental quantum cryptograms. a)** The semidefinite programming framework extracts a secure region of operation (turquoise) as a function of errors and losses. Our experimental performance $e_m = 0.0328 \pm 0.0001; l_m = 0.2239 \pm 0.015$ is indicated by the blue dot, and lies within the secure region. Error bars propagate poisson errors on coincidence counts. **b)** The dishonest success probability $p_d$ (green, upper bound) and honest success probability $p_h$ (red, lower bound) are displayed as a function of the number of quantum states $N$ required to verify one bit of the cryptogram. These are derived using a Chernoff bound argument [14]. As an example, an experimental token containing $\lambda = N = 4.2 \cdot 10^6$ quantum states (vertical blue dashed line) achieves an honest success probability very close to $p_h \sim 1$ and a dishonest success probability $p_d = 5.9 \cdot 10^{-45}$.

The numerical primal optimal values $\{e^{(\text{primal},1)}\}$ of Eq. (14) are plotted in Supplementary Figure 1.a as a function of loss tolerance $l$. Following the methods from Supplementary Note 1 A, we derive the dual problem associated with Eq. (14) to prove that $e^{(\text{primal},1)}$ provides a tight upper bound on the cheating probability. This problem can be written as:

$$\begin{aligned} \max\ & [\text{Tr}[X_1] + l \cdot (x_2 + x_3)] \\ \text{s.t.}\ & [\mathbb{1}_9 \otimes X_1 + L_0^\dagger \cdot x_2 + L_1^\dagger \cdot x_3 + (E_1 - E_0)^\dagger \cdot x_4 - E_0^\dagger] \le 0 \\ & \text{where } X_1 \text{ is a hermitian } 2 \times 2 \text{ matrix} \\ & x_2, x_3, x_4 \in \mathbb{R}, \end{aligned} \tag{15}$$

and its numerical optimal dual value $e^{(\text{dual},1)}$ indeed satisfies $e^{(\text{primal},1)} = e^{(\text{dual},1)}$. Note that the error and loss operators are hermitian, i.e. $L_x^\dagger = L_x$ and $E_x^\dagger = E_x$.

### B. For $N \to \infty$

In this section we show that, when $N \to \infty$, a malicious party does not gain any advantage in correlating the $N$ states in the quantum token (i.e., that the single-state security bounds derived in Supplementary Note 3 A still hold). Following the exponential de Finetti arguments from [15], it is sufficient to argue that, since our quantum token state is symmetric under arbitrary re-ordering of the $N$ quantum states, the individual states are well approximated by a mixture of independent and identically distributed states.

The security analysis based on semidefinite programs is convenient for $N = 1$ quantum states, and for proving that the resulting cheating strategy is indeed optimal. In our particular case, one can also derive an analytical expression that fits the optimal cheating strategy derived in Supplementary Note 3 A:

$$e^{(\text{primal},N)} = e^{(\text{dual},N)} = -\frac{1}{4+2\sqrt{2}} \cdot l + \frac{1-\frac{p_m}{2}}{8+4\sqrt{2}} \tag{16}$$

which is a function of losses $l$ and multiphoton emission probability $p_m$. We can therefore easily determine how many errors a malicious Client has to introduce in order to comply to a certain constraint on losses and still double spend. Equivalently, through simple inversion of this equation, we can determine the amount of losses needed for successful cheating given the amount of declared errors $e$:

$$l^{(\text{primal},N)} = l^{(\text{dual},N)} = \Big[-(4+2\sqrt{2}) \cdot \frac{e}{1-\frac{p_m}{2}} + \frac{1}{2}\Big] \cdot (1-\frac{p_m}{2}) = -(4+2\sqrt{2}) \cdot e + \frac{1}{2} \cdot (1-\frac{p_m}{2}) \tag{17}$$

Eq. (16) and Eq. (17) describe the secure region from Supplementary Figure 1.a. For simplicity, we define a new parameter $\mathcal{M}(e,l)$, which indicates the overall amount of *mishaps* (i.e. any combination of errors and losses) a TTP might receive from a malicious party. By upper bounding this expression, we can easily specify the secure region:

$$\mathcal{M}(e,l) = (4+2\sqrt{2}) \cdot e + l \leq \frac{1}{2} \cdot (1-\frac{p_m}{2}) \tag{18}$$

### C. For $N$ finite

Since $N$ will be finite in a realistic implementation, it is necessary to study the effect of finite-length statistics on the honest and dishonest success probabilities $p_h$ and $p_d$, respectively. A malicious party may indeed successfully cheat by introducing fewer losses or errors than the expected asymptotic values displayed in Supplementary Figure 1.a. We will make use of Chernoff-Hoeffding inequalities from Supplementary Note 1 C to bound this probability.

#### 1. *Honest success probability*

While the TTP allows for a certain amount of losses and errors, in order for the protocol to work in a realistic, i.e. imperfect scenario, there is still some probability, that the honest parties actually introduces more than the expected number of errors and/or losses, i.e. $\mathcal{M}(e_h^{\text{act}}, l_h^{\text{act}})$. We denote the probability that this occurs as $p_h^{\text{fail}}$. Following Supplementary Note 1 C, it is possible to upper bound $p_h^{\text{fail}}$ as:

$$p_h^{\text{fail}} = P\left[\mathcal{M}(e_h^{\text{act}}, l_h^{\text{act}}) \geq (1+\delta_h) \cdot \mathcal{M}(e_h, l_h)\right] \leq \exp\left(-\frac{(\delta_h)^2}{3} \cdot \mathcal{M}(e_h, l_h) \cdot N\right) \tag{19}$$

for some $\delta_h$. The honest success probability $p_h$ of the protocol is then defined as the probability that the protocol does *not* abort when it is followed honestly. Therefore it can be expressed as:

$$p_h = 1 - p_h^{\text{fail}} \geq 1 - \exp\left(-\frac{(\delta_h)^2}{3} \cdot \mathcal{M}(e_h, l_h) \cdot N\right) \tag{20}$$

As is apparent, the correctness increases exponentially with $\delta_h^2$ and $N$. However, since the TTP also has to allow for more errors and losses with increasing $\delta_h$, they are more vulnerable to malicious parties. We thus need to ensure that:

$$\mathcal{M}(e_h, l_h) \cdot (1+\delta_h) < \frac{1}{2} \cdot \left(1-\frac{p_m}{2}\right). \tag{21}$$

This inequality upper bounds the allowed value of $\delta_h$ without jeopardizing the information theoretical security of the protocol.

### 2. Dishonest success probability

Similarly, it might be possible for a cheating party to introduce fewer errors and losses than expected from the theoretical security proof. Following Supplementary Note 1 C, we upper bound this probability $p_d$ as:

$$p_d = P\left[\mathcal{M}(e_d^{\text{act}}, l_d^{\text{act}}) \leq (1-\delta_d)\cdot\frac{1}{2}\cdot(1-\frac{p_m}{2})\right] \leq \exp\left(-\frac{(\delta_d)^2}{2}\cdot\frac{1}{2}\cdot(1-\frac{p_m}{2})\cdot N\right) \tag{22}$$

for some $\delta_d$. Using Eq. (21), we must then ensure that:

$$\frac{1}{2}\cdot\left(\left(1-\frac{p_m}{2}\right)\cdot(1-\delta_d)\right) \geq \mathcal{M}(e_h, l_h)\cdot(1+\delta_h). \tag{23}$$

Since this is the amount of mishaps the TTP allows for in order to assure the correctness of the protocol.

## Supplementary Note 4. SECURITY OF THE CRYPTOGRAM

In our protocol, we use an *i.t.-secure MAC* to compute the measurement basis string for the quantum token $|P\rangle$. I.e., we measure $|P\rangle$ according to $MAC(C_i, M_i)$, where $C_i \in C$ is a preshared key of the Client with the TTP, and $M_i$ is the Merchant's ID where the payment token is spent. This facilitates the security of the quantum channels as well as the classical channels.

To guarantee a decent level of security, one has to fare the number of quantum states $N$ for a single payment token (see Supplementary Note 3) with the number of merchants $|M|$ and the size of the output tag $|\mathcal{T}|$. In the case of a *1-time-secure* function, we assume

$$\forall t \in \mathcal{T} \text{ that } |\mathcal{T}| = \sqrt{|\mathcal{K}|} = \sqrt{|C|} \ggg |M|$$

for a single authentication tag. The probability $p_t$ of forging the output of $MAC(C_i, M_i)$ should be similarly low as the *dishonest success probability* $p_d$ for a given sub-token size $N$ (see Supplementary Note 3, Supplementary Figure 1).

Thus, we choose $p_d \sim p_t \Rightarrow p_d \sim \frac{|M|}{\sqrt{|C|}} \sim \frac{1}{\sqrt{|C|}}$, and the overall the token length $\lambda = N\cdot\log_2|C|$.
We stress that this also holds for *n-time-secure* constructions (see Supplementary Note 2 D), just that $|C|$ needs to be chosen accordingly.

## Supplementary Note 5. INTUITION ABOUT POTENTIAL ATTACKS ON UNTRUSTED CHANNELS

In the following we will discuss the potential attacks our protocol protects against, and en passant explain which part of our scheme serves which precise purpose. Note that the following is only an intuitive explanation of the rigorous security proof provided in the previous section.

### A. Compromising classical channels

We have two classical channels in our protocol, namely `CH2` (Client $\rightarrow$ Merchant) and `CH3` (Merchant $\rightarrow$ TTP) in FIG. 1 of the main text. Since both of them are untrusted, it is possible for a malicious third party to intercept them and modify the cryptogram $\kappa(C, M_i, |P\rangle)$ towards another merchant $M_i'$ on `CH2` or change the merchant's Id $M_i$ towards another merchant's $M_i'$ on `CH3`.

**CH2:** To be accepted by the TTP, the attacker has to find another $\kappa'(C, M_i', |P\rangle)$ for the Client's secret $C$ that commits the purchase to another Merchant $M_i'$. This is impossible for two reasons:

1.) the attacker would need to determine $C$ from the Client to calculate a second measurement bases $m' = MAC(C, M_i')$, which is supposed to be securely distributed between Client and TTP. It is impossible to determine $C$ from $\kappa$ as the function of the measurement basis $MAC(C, M_i)$ is information-securely irreversible – and its output is additionally hidden in the quantum measurement and therefore unknown to the attacker.

2.) even if the attacker would have access to $C$ for some reason – e.g. by accessing the Client's memory – , he would require the classical description of quantum token $|P\rangle$, since it is already measured and quantum measurements are destructive. However, the classical description is only known by the TTP and never communicated.

**CH3:** To change $M_i$ that is communicated together with $\kappa$, the attacker has to find $M_i'$ that generates the same measurement bases $m' = MAC(C, M_i')$ that was used to generate $\kappa$. To do so, he would need access $C$, which is supposed to be securely distributed between Client and TTP. However, even if he would have access, the chances of finding a collusion such that $MAC(C, M_i) = MAC(C, M_i')$ for a given $C$ are exponentially low due to the information-theoretic nature of the MAC function.

If the Merchant requires instant notification of the payments acceptance, however, this channel requires authentication s.t. the Client could not alter this message.

Please note, that both attacks can be performed by a malicious Merchant as well – who has access to both channels and is supposed to be untrusted – but fail for the same reason.

### B. Compromising the quantum channel

A significant advantage of our scheme is that it is preferable but *not necessary* to authenticate the quantum channel used to distribute $|P\rangle$. Let us suppose that a malicious party intercepts the quantum states $|P\rangle$ and sends their own quantum states $|P'\rangle$ to the Client instead.
After the Client measures $|P'\rangle$ in the basis $MAC(C, M_i)$, they will hold the cryptogram $\kappa' = \kappa(C, M_i, |P'\rangle)$. If $\kappa'$ reaches the TTP, the transaction will be declined since $\kappa(C, M_i, |P'\rangle) \neq \kappa(C, M_i, |P\rangle)$ (within the error/loss tolerance allowed by the security analysis). This means, that the Client as well as the TTP will be able to detect that the quantum states have been tampered with and that precautions should be taken.
Another possible cheating strategy is for the malicious party to use the quantum token $|P\rangle$ themself and measure it in another basis than the Client had intended. However, the malicious party does not know $C$, since it was securely distributed only between Client and TTP, and is thus unable to determine any measurement basis $m_j$ that will be accepted for the Merchant that they choose.

### C. Compromising both channels simultaneously

Let us now suppose that a malicious party intercepts $|P\rangle$ on the quantum channel, replaces it with another quantum token $|P'\rangle$, and waits for the honest Client to send the resulting $\kappa'$ on the classical channel. If the Client would measure $|P'\rangle$ in a basis that is dependent on $C$ in a simple way, e.g. $m_i = M_i \oplus C$ then the malicious party gains knowledge of $C$, and can then substitute the Client's identity in multiple transactions. This is why we use a MAC instead: even if the malicious third party gets hold of $\kappa'$ and, by knowing $|P'\rangle$, deduces the measurement basis $m_i$, they are unable to retrieve $C$, because of the information theoretically secure nature of the used MAC, i.e. because the number of collision ensures that no cheating strategy would be better than guessing. Thus again, the TTP (and subsequently the Client) realise that something is wrong, while the secret Client token $C$ remains hidden.

Depending on the nature of the MAC the token $C$ may resist a certain amount of failures, before it has to be exchanged.

## Supplementary Note 6. EXPERIMENTAL DETAILS

This section is dedicated to our protocol performance and setup characterization. Supplementary Table 1 presents the measured transmission and corresponding loss rate of various setup components, while Supplementary Table 2 details the setup characterization and security performance.

| Component | Transmission | Losses |
|---|---|---|
| Fibre link | 93.4 ± 1.5 % | 6.9 ± 1.5 % |
| Client setup | 89.82 ± 0.57 % | 10.18 ± 0.57 % |
| Detector 1 | 93.34 ± 0.75 % | 6.66 ± 0.75 % |
| Detector 2 | 92.51± 0.78 % | 7.49 ± 0.78 % |
| Overall | 77.6 ± 1.5 % | 22.4 ± 1.5 % |

Supplementary Table 1: **Transmission and corresponding losses of the experimental setup.**

| | Parameter name | Variable | Value |
|---|---|---|---|
| Experimental results | Losses | $l_m$ | 22.4 ± 1.5 % |
| | Errors H/V | $e_m^{H/V}$ | 1.4491 ± 0.0083 % |
| | Errors +/- | $e_m^{+/-}$ | 3.278 ± 0.013 % |
| | Mishaps | $\mathcal{M}(e_m^{+/-}, l_m)$ | 0.448 ± 0.016 |
| | Sub-token length | $N$ | 4 482 440 ± 1600 |
| | Q-bit rate | $1/s$ | 14 298 ± 6 Hz |
| | Correlation function | $g_2(0)$ | 0.03010 ± 0.00014 |
| | Multiphoton emission | $p_m$ | 6.02 ± 0.28 % |
| SDP | Dishonest errors | $e_d(l_m)$ | 3.82 ± 0.22 % |
| | Dishonest losses H/V | $l_d(e_m^{H/V})$ | 38.600 ± 0.058 % |
| | Dishonest losses +/- | $l_d(e_m^{+/-})$ | 26.111 ± 0.090 % |
| C-H bounds | Allowed mishaps | $\mathcal{M}(e, l)$ | 0.4849 |
| | Tolerance correctness | $\delta_h$ | 0.031 |
| | Honest success probability | $p_h$ | $\sim 1$ |
| | Tolerance cheating | $\delta_d$ | 0.01 |
| | Dishonest success probability | $p_d$ | $5.911 \pm 0.088 \cdot 10^{-45}$ |

Supplementary Table 2: **Setup characterization and protocol performance.**

## Supplementary References


[1] Watrous, J. Semidefinite programming. *Theory of Quantum Information (notes from Fall 2011)* (2011). URL `https://web.archive.org/web/20220521201106/https://cs.uwaterloo.ca/~watrous/TQI-notes/`.

[2] Vandenberghe, L. & Boyd, S. Semidefinite programming. *SIAM Review* **38**, 49–95 (1996). URL `https://doi.org/10.1137/1038003`.

[3] Molina, A., Vidick, T. & Watrous, J. Optimal counterfeiting attacks and generalizations for wiesner's quantum money. vol. 7582 of *Lecture Notes in Computer Science* (Springer, 2013). URL `https://doi.org/10.1007/978-3-642-35656-8_4`.

[4] Chernoff, H. A Measure of Asymptotic Efficiency for Tests of a Hypothesis Based on the sum of Observations. *The Annals of Mathematical Statistics* **23**, 493 – 507 (1952). URL `https://doi.org/10.1214/aoms/1177729330`.

[5] Hoeffding, W. *Probability Inequalities for sums of Bounded Random Variables*, 409–426 (Springer New York, New York, NY, 1994). URL `https://doi.org/10.1007/978-1-4612-0865-5_26`.

[6] Mitzenmacher, M. & Upfal, E. *Probability and Computing: Randomized Algorithms and Probabilistic Analysis* (Cambridge University Press, 2005). URL `https://doi.org/10.1017/CBO9780511813603`.

[7] Katz, J. & Lindell, Y. *Introduction to Modern Cryptography, Second Edition* (Chapman and Hall/CRC, 2014).

[8] Stinson, D. R. *Cryptography: Theory and practice*. Discrete mathematics and its applications (Chapman & Hall/CRC, Londen [etc.], 2006), 3rd ed. edn.

[9] Wegman, M. N. & Carter, J. New hash functions and their use in authentication and set equality. *Journal of Computer and System Sciences* **22**, 265–279 (1981).

[10] Gilbert, Edgar N and MacWilliams, F Jessie and Sloane, Neil JA. Codes which detect deception. *Bell System Technical Journal* **53**, 405–424 (1974). URL `https://ieeexplore.ieee.org/document/6770988`.

[11] Fak, V. Repeated use of codes which detect deception (Corresp.). *IEEE Transactions on Information Theory* **25**, 233–234 (1979).

[12] Rosenbaum, Ute. A lower bound on authentication after having observed a sequence of messages. *Journal of Cryptology* **6**, 135–156 (1993). URL `https://link.springer.com/article/10.1007/BF00198462`.

[13] Brańczyk, A. M., Ralph, T. C., Helwig, W. & Silberhorn, C. Optimized generation of heralded fock states using parametric down-conversion. *New J. Phys.* **12**, 063001 (2010). URL `https://doi.org/10.1088/1367-2630/12/6/063001`.

[14] Dubhashi, D. P. & Panconesi, A. *Concentration of Measure for the Analysis of Randomized Algorithms* (2009). URL

https://doi.org/10.1017/CBO9780511581274.
[15] Renner, R. Security of quantum key distribution (2005). URL https://arxiv.org/abs/quant-ph/0512258.
